# Identifications of Genes Involved in ABA and MAPK Signaling Pathways Positively Regulating Cold Tolerance in Rice

**DOI:** 10.3390/plants14040498

**Published:** 2025-02-07

**Authors:** Guohua Ding, Zhugang Li, Zubair Iqbal, Minghui Zhao, Zhibo Cui, Liangzi Cao, Jinsong Zhou, Lei Lei, Yu Luo, Liangming Bai, Guang Yang, Rongsheng Wang, Kun Li, Xueyang Wang, Kai Liu, Mingnan Qu, Shichen Sun

**Affiliations:** 1 Institute of Crop Cultivation and Tillage, Heilongjiang Academy of Agricultural Sciences/Heilongjiang Rice Quality Improvement and Genetic Breeding Engineering Research Center, Harbin 150086, China; hucheng229@163.com (G.D.); lizhugang@163.com (Z.L.); 15304608296@163.com (L.C.); zhoujinsong168@163.com (J.Z.); 18646085786@163.com (L.L.); luoyusun@126.com (Y.L.); bailiangming1970@163.com (L.B.); ouyanghuiru@163.com (G.Y.); 2 Heilongjiang Academy of Agricultural Sciences/Northeast Center of National Salt-Alkali Tolerant Rice Technology Innovation Center, Harbin 150086, China; rshwang@haas.cn (R.W.); likun.steven@163.com (K.L.); hljsnkywxy@163.com (X.W.); liukailouis@163.com (K.L.); 3 Jiangsu Key Laboratory of Crop Genomics and Molecular Breeding, College of Agriculture, Yangzhou University, Yangzhou 225009, China; zubairiqbal@yzu.edu.cn; 4 Design and Germplasm Innovation/Collaborative Innovation Center for Genetic Improvement and High Quality and Efficiency Production of Northeast Japonica Rice in China, Shenyang Agricultural University, Shenyang 110161, China; mhzhao@syau.edu.cn (M.Z.); cuizb@syau.edu.cn (Z.C.); 5 Nanfan Research Institute, Chinese Academy of Agricultural Sciences, Sanya 572024, China

**Keywords:** cold stress, ABA signaling, MAPK signaling, transcriptional regulation, integrative analysis

## Abstract

Cold stress (CS) significantly impacts rice (*Oryza sativa* L.) growth during seedling and heading stages. Based on two-year field observations, this study identified two rice lines, L9 (cold stress-sensitive) and LD18 (cold stress-tolerant), showing contrasting CS responses. L9 exhibited a 38% reduction in photosynthetic efficiency, whereas LD18 remained unchanged, correlating with seed rates. Transcriptome analysis identified differentially expressed genes (DEGs) with LD18 showing enriched pathways (carbon fixation, starch/sucrose metabolism, and glutathione metabolism). LD18 displayed dramatically enhanced expression of MAPK-related genes (*LOC4342017*, *LOC9267741*, and *LOC4342267*) and increased ABA signaling genes (*LOC4333690*, *LOC4345611*, and *LOC4335640*) compared with L9 exposed to CS. Results from qPCR confirmed the enhanced expression of the three MAPK-related genes in LD18 with a dramatic reduction in L9 under CS relative to that under CK. We also observed up to 66% reduction in expression levels of the three genes related to the ABA signaling pathway in L9 relative to LD18 under CS. Consistent with the results of photosynthetic efficiency, metabolic analysis suggests pyruvate metabolism, TCA cycle, and carbon metabolism enrichment in LD18 under CS. The study reveals reprogramming of the carbon assimilation metabolic pathways, emphasizing the critical roles of the key DEGs involved in ABA and MAPK signaling pathways in positive regulation of LD18 response to CS, offering the foundation toward cold tolerance breeding through targeted gene editing.

## 1. Introduction

Temperature stands as a pivotal environmental factor shaping plant distribution in terrestrial ecosystems [1]. Globally, cold-induced crop losses pose a significant challenge. With the continuous degradation of our ecological environment, cold has emerged as a growing threat to plant life. Cold stress (CS) compromises cell membrane integrity, triggering the production of reactive oxygen species (ROS) and other detrimental compounds, ultimately impeding plant growth and yield formation [2,3,4]. Numerous studies have investigated rice’s molecular response to CS, probing its physiological and ecological characteristics [5,6]. Cold significantly inhibits rice’s growth index and the physiological enzyme activities, a significant inhibition by prior cold treatments [7], hinting at induced cold tolerance. Under cold conditions, various genes have been observed to undergo an upregulation [8]. These genes encode proteins involved in plant cold tolerance’s sensing and signal transduction processes, stimulating the synthesis of osmotic regulators [9], enhancing antioxidant enzyme activity [10], and improving cell membrane fluidity [8]. This enhances the plant’s resilience to CS [8]. Therefore, unraveling the intricate process and mechanisms underlying the perception and transmission of cold signals and stimulating cold tolerance in plants hold great theoretical and practical significance. It contributes to a deeper understanding of cold tolerance mechanisms in plants and expedites the molecular breeding process for cold-tolerant crops.

Previous studies underscore the significance of transcriptional regulation in plant responses to cold injury [11]. The CBF/DREB-dependent signaling pathway drives cold tolerance in *Arabidopsis thaliana* and rice [12]. Notably, activating the calcium signaling pathway post-accumulation of cold tolerance characteristics reveals evolutionarily conserved genes crucial to plant adaptation [13]. Extensive research showcased the significant enhancement of cold tolerance in *japonica* rice by overexpressing the key QTL gene, *COLD1*, associated with sensing cold stress. Conversely, functional deletion mutants and antisense gene lines of *COLD1* exhibited susceptibility to cold damage [6]. Additionally, genomic investigations identified three genes (*Os01g55510*, *Os01g55350*, and *Os01g55560*) in chromosome 1 closely linked to cold tolerance, revealing the intricate nature of plant responses [14]. Several transcription factors, such as *MYBS3*, *OsWRKY71*, and *OsWRKY76*, have emerged as pivotal players in rice’s cold tolerance [15,16,17]. Recent studies have notably highlighted the role of calcium-dependent protein kinases in rice’s cold tolerance [18]. These findings serve as crucial theoretical references, laying the scientific basis for deeper exploration and understanding of rice’s molecular mechanisms and regulatory networks governing cold tolerance.

Recently, advancements in omic analysis, encompassing transcriptome, metabolism, and proteomics approaches, have emerged as crucial tools for identifying potential biomarkers in both abiotic and biotic stress in higher plants [19]. Numerous studies have delved into rice transcriptome profiling using RNA-seq technology to comprehend molecular pathways and compare transcriptomes [20,21,22,23,24,25]. Through CS evaluation across two consecutive years, we identified two distinct rice genotypes: LD18, categorized as highly tolerant, and L9, classified as highly susceptible to CS. The highly tolerant genotype LD18 exhibited survival for up to 25 d under CS of 10 °C. Therefore, this investigation integrates transcriptome and non-targeted metabolism analysis of these contrasting genotypes, aiming to identify promising genes and unveil novel insights into diverse gene expressions and pathways pivotal in conferring cold tolerance in rice.

## 2. Results

**Figure 1:**
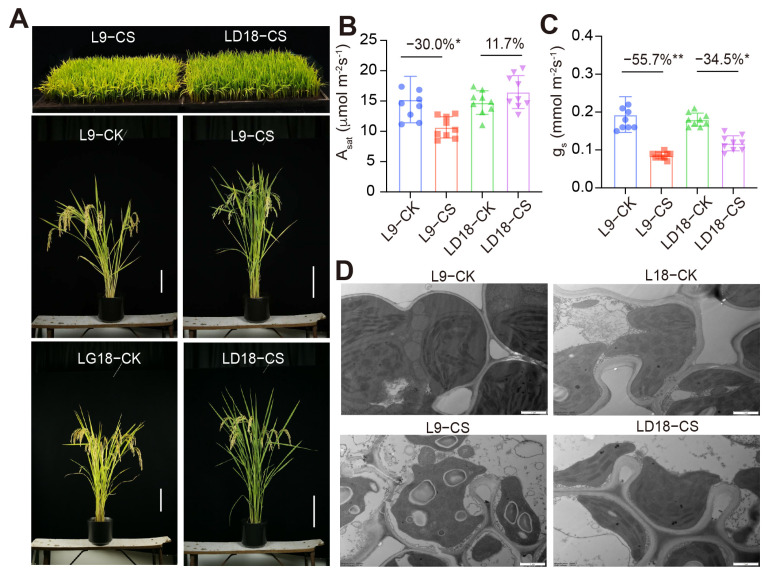
Performance of LD18 and L9 rice lines exposed to 10 d CS at both seedling and heading stages. (**A**) Images of potted-grown plants at the seedling stage (upper panel) and heading stage (lower panels). (**B**,**C**) Photosynthetic rates and stomatal conductance under saturated light conditions in the leaves of LD18 and L9 during the heading stage. Vertical bars represent means ± S.E. (*n* = 9). Percentage differences in CS compared with the CK for each rice line are shown. Symbols “*,” and “**” indicate significant differences at *p* < 0.05 and *p* < 0.01, respectively, based on Student *t*-test analysis. (**D**) Chloroplast structure images of LD18 and L9 rice lines exposed to either CK or CS.

This study encompassed the screening of 128 rice lines sourced from northern China, conducted over two consecutive years to assess their response to CS (Supplementary Data S1). From this screening, two distinct rice lines emerged based on their empty seed rates: L9, classified as a CS-sensitive rice line, and LD18, characterized as a CS-tolerant variant. Results revealed a marked distinction in phenotypic responses between L9 and LD18 (Figure 1A). As expected, CS induced a 30% inhibition in photosynthetic rates in L9 during the heading stage, while marginal differences were observed in LD18 due to CS effects (Figure 1B). Moreover, stomatal conductance exhibited a more pronounced decline in L9 compared with LD18 (Figure 1C), indicating that the inhibitory effects of CS on these rice lines were attributed to both impaired photosynthetic machinery and stomatal limitations. Correspondingly, results from transmitted electron microscopy revealed substantial changes in the chloroplast structure of L9 under CS, whereas the chloroplast structure of LD18 exhibited less change under the same conditions (Figure 1D).

Regarding spike traits, conspicuous detrimental effects of CS on spike filling during the heading stage were observed in both rice lines. However, these effects were notably more pronounced in L9 compared with LD18, whereas no discernible impact on spike length was noted for both lines (Figure S1A,B). Furthermore, as expected, the percentage values of the entire seed declined by 38% in L9 under CS exposure. At the same time, LD18 showed no significant difference, resulting in an eight-fold increase in empty seed percentage values for L9 and a four-fold increase for LD18 (Figure S1C,D). Interestingly, CS induced a significant rise in non-faired tillering in both rice lines (Figure S1E).

To delve deeper into the impact of CS on antioxidant and oxidoreductase activity in two rice lines, we measured the enzymatic features of eight compounds: GR (glutathione reductase), soluble protein content, SOD (superoxide dismutase), GSH-Px (reduced glutathione), CAT (catalase), POD (peroxidase), AS (asparagine synthase), and GS (glutamine synthetase) (Figure S2A–H). Our findings revealed inhibited soluble protein content, CAT, and AS in both rice lines under CS. Specifically, CAT and AS exhibited a more pronounced reduction in L9 compared with LD18 (Figure S2E,G). While GR, SOD, and POD were enhanced in both rice lines due to CS. Remarkably, there was a dramatic increase in GSH-Px and GS levels in L9, whereas no significant difference was observed in LD18 under similar CS conditions (Figure S2D,H).

Considering the diminished photosynthetic activity observed in L9 under CS, we investigated the activities of enzymes associated with carbon assimilation and energy metabolism. Our findings reveal distinctive enzymatic responses. Among the enzymes analyzed, only NAD-MDH presented a notable decrease of 34% in L9, whereas, in LD18, it exhibited a three-fold increase in exposure to CS (Figure S3A). Contrastingly, NADP-MDH demonstrated an 11.4% increase in L9 and a more pronounced 27.5% increase in LD18 (Figure S3B). Interestingly, NAD-ME levels increased by 93% in L9 but declined by 47% in LD18 (Figure S3C). Additionally, while NADP-ME exhibited a 12% increase in L9, no significant variation was observed in LD18 under CS (Figure S3D).

**Figure 2:**
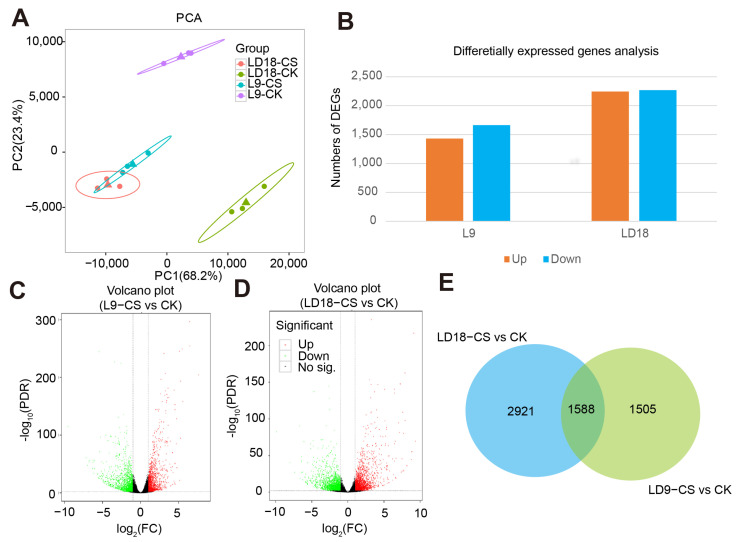
Transcriptome analysis in LD18 and L9 under CS during the heading stage. (**A**) Principal component analysis. (**B**) Statistical analysis of the no. of DEGs in L9 and LD18 exposed to CS. (**C**,**D**) Volcano plot representing DEGs in L9 under CS relative to CK and DEGs in LD18 under CS relative to CK. (**E**) Overlapped DEGs between L9 and LD18 under CS relative to CK.

The above evidence suggests that photosynthetic assimilation reduction in L9 is related to impaired photosynthetic machinery, stomatal limitations, inhibited antioxidant enzymes, and photosynthetic enzyme activities. We conducted an integrative analysis of transcriptome and non-targeted metabolism to unveil the key differentially expressed genes (DEGs) contributing to these varying performances between the rice lines (L9 and LD18) under CS. Our analysis of transcriptome data revealed that, on average, GC content and Q30 in 12 samples were 0.52 and 94%, respectively (Table S2). Across LD18 and L9 samples, the number of clean reads was 21.37 million (Table S2). PCA results indicated that PC1 and PC2 contributed 68.2% and 23.4% to transcriptome dataset variation (Figure 2A). Within L9, 1425 genes were upregulated and 1528 downregulated under CS, whereas LD18 exhibited 2495 upregulated and 2472 downregulated DEGs in CS relative to under CK (Figure 2B–D; Supplementary Data S2). There were 1588 overlapped DEGs between LD18 and L9 under CS relative to CK (Figure 2E).

Gene Ontology (GO) analysis of DEGs revealed significant enrichment in specific biological processes for both L9 and LD18 under CS compared with CK. In L9, enriched processes included cinnamic acid biosynthetic pathways, L-phenylalanine catabolism, trehalose biosynthetic, protein folding, glutamine metabolic pathways, and chloroplast–nucleus signaling (Figure S4A). Contrastingly, in LD18, enriched processes encompassed photosynthesis, regulation of jasmonic acid-mediated signaling, cation transmembrane transport, chlorophyll biosynthetic, protein refolding, stress response, and trehalose biosynthesis under CS relative to CK (Figure S4B).

**Figure 3:**
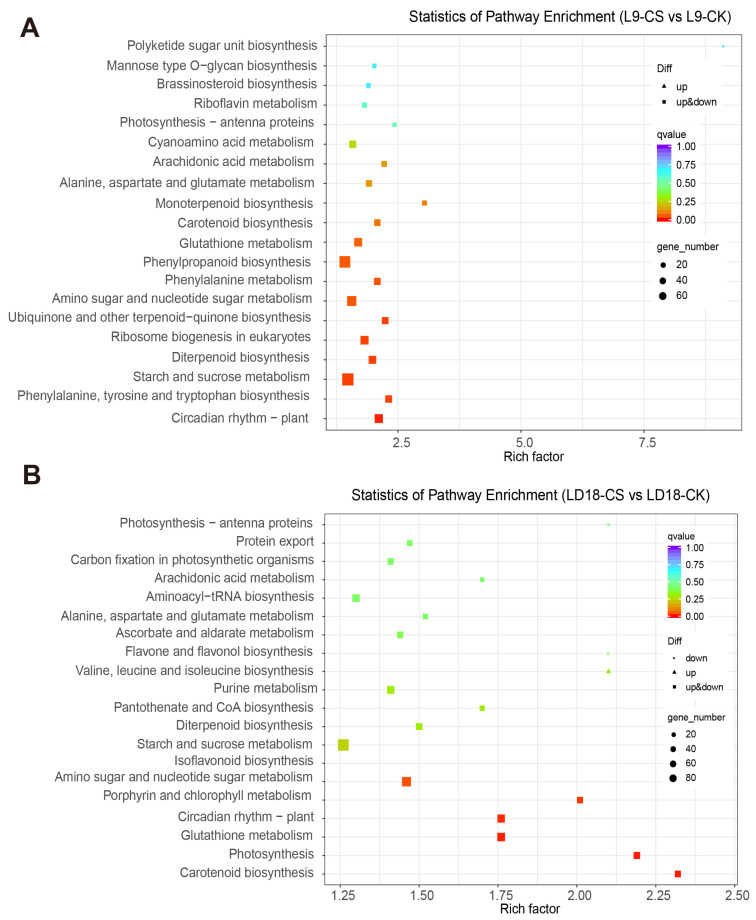
KEGG analysis was performed on DEGs in the leaves of LD18 and L9 exposed to CS. (**A**) KEGG analysis on the DEGs in L9 under CS compared with CK. (**B**) KEGG analysis on the DEGs in LD18 under CS compared with CK.

The KEGG analysis revealed significant enrichments in DEGs in response to CS in both L9 and LD18 rice lines compared with CK. In L9 under CS, pathways such as brassinosteroid biosynthesis, riboflavin metabolism, photosynthesis-antenna proteins, arachidonic acid metabolism, glutathione metabolism, and phenylalanine metabolism showed significant enrichment among the DEGs (Figure 3A). Conversely, in LD18 under CS, pathways including carbon fixation in photosynthetic organisms, arachidonic acid metabolism, flavone and flavanol biosynthesis, starch and sucrose metabolism, and glutathione metabolism exhibited significant enrichment within the DEGs (Figure 3B).

**Figure 4:**
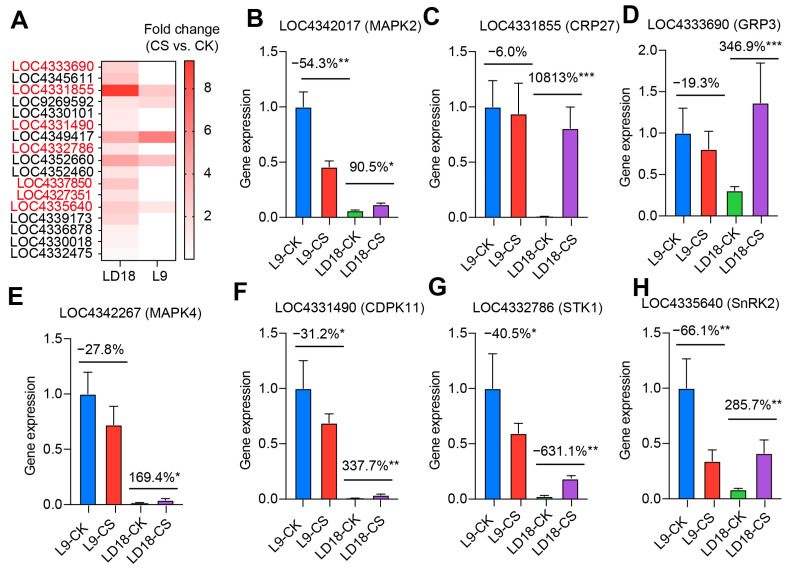
qPCR validation of upregulated DEGs in LD18 due to CS during the heading stage. (**A**) Fold change of 17 promising DEGs, representing the top 1% DEGs from the overlapped set between LD18 and L9 exposed to CS relative to CK. (**B**–**H**) Expression profiles of individual genes: LOC4342017 (*MPK2*), LOC4331855 (*CRP27*), LOC4333690 (*GRP3*), LOC4342267 (*MAPK4*), LOC4331490 (*CDPK* 11), LOC4332786 (*STK1*), and LOC4335640 (*SnRK2*). Vertical bars represent means ± S.E. (*n* = 3). Percentage differences in CS against CK for each rice line. Symbols “*”, “**”, and “***” indicate significant differences at *p* < 0.05, *p* < 0.01, and *p* < 0.001, respectively, based on Student t-test analysis.

To identify the promising DEGs governing the distinct responses to CS in LD18 and L9, we selected the top 1% DEGs from the overlapped dataset between these lines exposed to CS. Validation of these DEGs was performed using qPCR (Figure 4A–H). The findings confirmed notable differences in the expression levels of the genes associated with the MAPK signaling pathway between LD18 and L9 under CS compared with their respective CK. Genes like LOC4342017 (*MPK2*), LOC9267741 (*MAPK1*), and LOC4342267 (*MAPK4*) exhibited a significant increase in LD18 but a remarkable decrease in L9 under CS (Figure 4B,E and Figure S5A). Similarly, genes related to the ABA signaling pathway, including LOC4333690 (*GRP3*), LOC4345611 (*RBCX1*), and LOC4335640 (*SnRK2*), showed a substantial reduction by up to 66% in L9, while these genes showed substantial enhancement in LD18 under CS compared with CK (Figure 4D,H and Figure S5B). Comparable trends were observed for other genes such as LOC4352660 (*CWM1*), LOC4331855 (*CRP27*), LOC4331490 (*CDPK11*), and LOC4332786 (*STK1*) (Figure 4C,F,G and Figure S5C).

**Figure 5:**
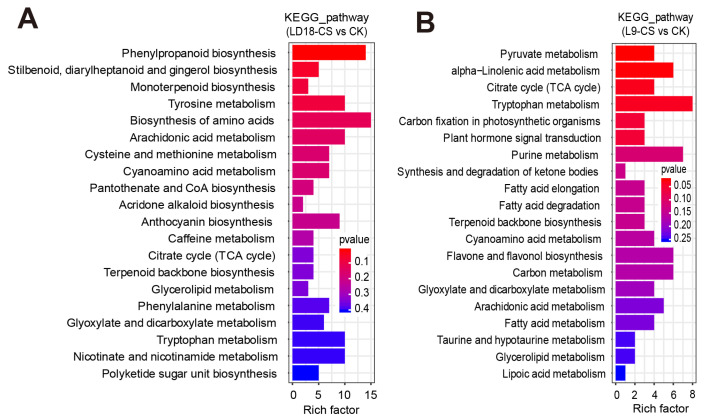
KEGG analysis on DAMs in the leaves of LD18 and L9 exposed to CS based on a non-targeted metabolism dataset. (**A**) KEGG analysis on DAMs in L9 under CS compared with CK. (**B**) KEGG analysis on DAMs in LD18 under CS compared with CK.

As indicated by transcriptome analysis, both photosynthesis and chlorophyll biosynthetic pathways are involved in CS in two rice varieties. These findings enable us to explore the changes in photosynthetic carbon metabolism in the two rice varieties exposed to CS. Based on the non-targeted metabolism analysis, we performed PCA, DAMs, and extreme DAMs analysis to step-by-step present our in-depth metabolic analysis, as shown in Figures S6 and S7. PCA revealed that L9, PC1, and PC2 accounted for 83.5% and 8.5% of the variance, respectively (Figure S6A). Correspondingly, for LD18, PC1 and PC2 accounted for 91.2% and 6.1% of variance, respectively (Figure S6B). Notably, specific metabolites exhibited a dramatic upregulation in L9 under CS compared with CK, including toluene-4-sulfonate, aloesin, and (6E)-8-Oxolinalool (Figure S6C). Detailed information on extreme DAMs is listed in Figure S7A,B. Moreover, KEGG analysis based on non-targeted metabolism data revealed significant enrichments in specific metabolic pathways for both LD9 and LD18 under CS relative to CK. In LD9 under CS, pathways such as phenylpropanoid biosynthesis, tyrosine metabolism, amino acids biosynthesis, arachidonic acid metabolism, anthocyanin biosynthesis, and glyoxylate and dicarboxylate metabolism were significantly enriched (Figure 5A). Conversely, in LD18 under CS, pathways like pyruvate metabolism, citrate cycle (TCA cycle), carbon fixation in photosynthetic organisms, flavone and flavanol biosynthesis, and carbon metabolism significantly enriched among the list of DAMs (Figure 5B).

**Figure 6:**
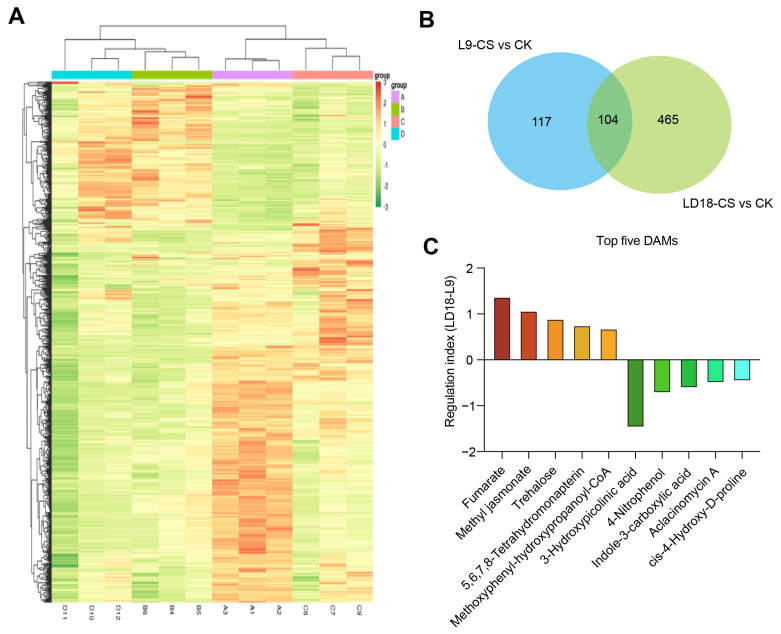
Identification of promising DAMs in L9 relative to LD18 due to CS effects. (**A**) Heatmap illustrating the DAMs in L9 and LD18 under both CS and CK conditions. (**B**) Overlapped DAMs between L9 and LD18 under CS relative to CK. (**C**) Regulation of DAMs between L9 and LD18 under CS relative to CK. The regulation index was calculated by subtracting the log2(FC) in LD18 from that in L9.

To further identify the key DAMs responsible for the distinct responses observed in L9 and LD18 exposed to CS, we performed a comprehensive analysis of overlapped DAMs. Our findings revealed 221 DAMs in L9 and 569 DAMs in LD18 when exposed to CS compared with CK (Figure 6A,B). In total, 104 DAMs overlapped between LD18 and L9 under these CS conditions (Figure 6B). Utilizing log2FC values, we calculated the regulation index, uncovering significant increases in fumarate, methyl jasmonate, trehalose, tetrahydromonapterin, and methoxyphenyl-hydroxypropyl-CoA, particularly in LD18, surpassing the increases observed in L9 exposed to CS (Figure 6C; Table S4). Conversely, 4-nitrophenol, indole-3-carboxylic acid, actinomycin A, and cis-4-hydroxy-D-proline were dramatically inhibited in both rice lines, with L9 revealing a more pronounced reduction compared with LD18 (Figure 6C).

**Figure 7:**
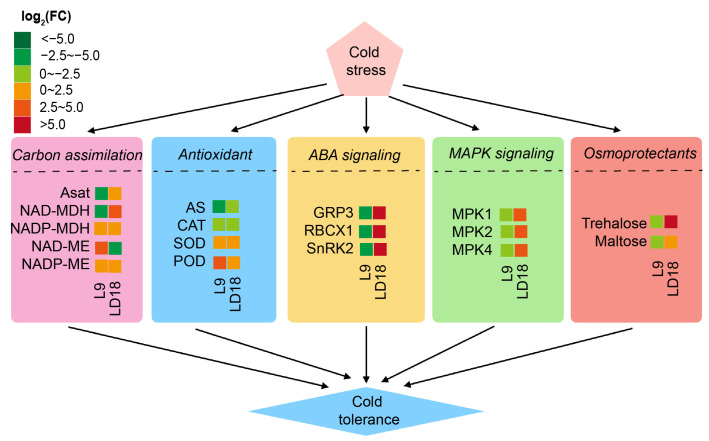
A summarized model illustrates the overall changes in biological and metabolic pathways in LD18 and L9 under CS conditions. The colors are scaled to the log2(FC) ranges in L9 (left cell) and LD18 (right cell).

In summary, our findings demonstrate that CS could induce significant reprogramming across various biological and metabolic pathways, notably impacting carbon assimilation, ROS scavenging via antioxidants, ABA signaling transduction, MAPK signaling pathway, and several osmoprotectant metabolites (Figure 7). Our observations suggest that the ABA and MAPK signaling pathways might play critical roles in the response to cold stress in both LD18 and L9 rice lines. These pathways are essential to understanding the plant’s response to cold stress.

## 3. Discussion

### 3.1. Physiology Responses to Cold Stress

Plants often encounter physiological disruptions when exposed to CS, significantly impacting their growth and developmental processes [1,26]. While initial exposure to low nonfreezing temperatures might trigger cold tolerance, sustained exposure becomes catastrophic. Chloroplasts, pivotal in photosynthetic processes, play a critical role in rice development. CS induces cold injury in rice leaves, hindering chlorophyll synthesis and reducing photosynthesis [27,28,29]. Photosynthesis is crucial in rice yield and is a prominent physiological CS process [30]. Our study mirrors this impact, showcasing the contrasting responses of cold-sensitive rice (L9) and cold-tolerant rice (LD18) to low temperatures (Figure 1B). LD18 exhibits marked resilience and adaptation to cold-induced damage compared with L9.

### 3.2. DEGs Involved in the Pathway of Photosynthesis

Consistent with the observed photosynthetic responses in L9 and LD18 (Figure 1B), our analysis revealed a significant enrichment of the photosynthetic pathway in the LD18 under CS across multiple metabolic pathways, as indicated by both GO and KEGG analyses. This enrichment underscores the crucial role played by an activated photosynthetic pathway in mitigating cold sensitivity in LD18. Recognized as the basis of life on earth, photosynthesis heavily relies on Rubisco, a pivotal enzyme in the photosynthetic process. It was noticed that the lessened performance of C4 plants under cold conditions might stem from possessing 60–80% less Rubisco on a total protein basis than C3 plants [31]. Increased Rubisco content has shown promise in mitigating cold stress and hastening recovery, as seen in maize [32]. In our study, we noted a three-fold increase in the transcript abundance of *RBCX1* (*LOC4345611*) in LD18 exposed to CS, whereas there was no significant change in L9. This suggests the critical role of this chaperone in facilitating Rubisco folding during the response to CS in rice. Such findings underscore the significance of proper Rubisco folding in enhancing photosynthesis during CS, potentially alleviating the cold effects. Notably, the orthologous gene of *RBCX1* (*AT4G04330*) in Arabidopsis exhibited dramatic stimulation under CS conditions, as indicated by the transcriptome analysis [33].

### 3.3. ABA Signaling in Cold Stress Response

The involvement of ABA in signaling cascades during cold stress is pivotal. Studies have indicated that cold stress triggers the accumulation of endogenous ABA in plants [34]. Additionally, exogenous ABA has been shown to enhance plants’ cold tolerance [35]. Microarray data analysis in a specific cold-tolerant rice variety unraveled the complicated crosstalk between the CBF and ABA-responsive element (ABRE) regulons, emphasizing the role of ABA signaling in rice cold tolerance [36]. Within this signaling pathway, sucrose non-fermenting 1 (SNF1)-related protein kinase 2s (SnRK2s) play key roles [37]. Our study validated these findings, observing that the expression levels of two SNF1-related protein kinase genes (*LOC4335640* and *LOC4339173*) were upregulated at least two-fold in LD18. In contrast, these genes remained unaltered or exhibited decreased expression in L9 under CS, as shown by both transcriptome analysis and qPCR validation (Table S3; Figure 4H). In the absence of ABA, SnRK2s are suppressed by PP2Cs, maintaining the inactive state of the ABA signaling pathway [38,39]. SnRK2s subfamily members are closely linked to regulating plant response to abiotic stress and abscisic acid (ABA) [40]. Upon exposure to ABA, PYR/PYL/RCAR cancels the PP2C-mediated inhibition of SnRK2s, and the released SnRK2s autophosphorylate and self-activate, activating or inhibiting various downstream targets. In Arabidopsis, it was reported that SnRK2.3/SRK2I plays a positive role in seed germination under cold stress conditions [37]. Our study identified that the two genes of SnRK2s were upregulated in the CS tolerance rice line, further supporting the critical roles of SnRK2 in CS in plants.

### 3.4. MAPK Signaling in Cold Stress Response

Mitogen-activated protein kinase (MAPK) cascades have been recognized for their pivotal roles in various aspects of plant biology, particularly in responding to CS [41]. Studies in Arabidopsis have highlighted the activation of MAPK genes such as *MEKK1*, *MKK2*, *MPK4*, and *MPK6* under CS [42], while two pathways can regulate CS. AtMEKK1-AtMKK2-AtMPK4 positively regulates cold stress, whereas AtMKK4/5-AtMPK3/6 negatively regulates cold stress [43]. Interestingly, when CS occurs, AtMPK3/6 can phosphorylate AtICE1 and AtMYB15, which induces AtICE1 fast degradation and represses AtMYB15′s binding affinity, which, in turn, attenuates AtCBF3 transcription. Our study aligns with these observations, revealing that the expression levels of several genes associated with the MAPK signaling pathway (e.g., *LOC9267741*, *LOC4342267*, and *LOC4342017*) were upregulated by up to 2.8-fold in CS-tolerant rice line LD18 under cold conditions. In contrast, these genes exhibited inhibition in L9 under CS, with reductions of 13% (Figure 4B,E and Figure S5A and Table S3). Among the three MAPK genes, *LOC4342267* is also annotated as OsMAPKKK11, which interacts with OsRLCK185 in the chitin signaling pathway and regulates rice immune response [44,45]. These findings suggest that CS triggers the induction of kinase molecules, enabling some cold tolerance rice varieties to adapt and survive adverse environments, probably through the chitin signaling pathway.

### 3.5. Metabolite’s Roles in ROS Scavenging During Cold Stress Response

CS instigates substantial metabolic changes and impacts various physiological properties in plants, notably activating the cold-responsive signaling network in rice. Trehalose is a non-reducing disaccharide sugar comprising two glucose molecules that serve as an osmoprotectant and play a crucial role in plant metabolism and signaling mechanisms [46]. Studies have shown that trehalose modulates diverse osmotic substances in tomato plants under CS [47]. In line with this, our findings indicate a significant accumulation of trehalose in CS-tolerant rice line LD18 compared with L9 (Figure 6C; Table S4). These observations underscore the pivotal role of trehalose in regulating CS tolerance responses in rice. This emphasizes the significance of trehalose in plant metabolism and in orchestrating crucial mechanisms vital for combating cold stress.

ROS are essential signaling molecules influencing plant growth, development, and responses to diverse stresses [48]. Studies have highlighted trehalose’s role in diminishing ROS production as a defensive response strategy during plant stress [49,50]. Trehalose is a promising antioxidant because it protects against oxidative stress and works with other antioxidants to protect against cellular damage by abiotic stress [51]. In this study, the levels of some antioxidant enzymes, including POD and SOD, in LD18 were significantly higher compared with those in L9 (Figure S2A–H). This elevation contributed to shielding rice leaves, fortifying cell membrane functionality, mitigating ROS-induced damage, and enhancing cold tolerance in plants.

### 3.6. Collaborative Roles of MAPK and ABA Signaling in CS

Under stress conditions, plants respond by inhibiting growth to conserve energy and maximize survival. Some kinases work together to regulate energy homeostasis. For example, AMPK (5′ AMP-activated protein kinase) and SnRK1 (Snf1-Related Kinase 1) are conserved heterotrimeric kinase complexes, and the kinase complexes play conserved and essential roles through inducing metabolic reprogramming and achieving energy homeostasis in plants and hence alter growth, development and stress responses in plants [52,53,54]. The disaccharide trehalose is used as an energy source and storage and transport molecule for glucose by T6P. At the same time, recent studies have shown that T6P inhibits the activity of SnRK1 in both monocots and dicots [55,56], suggesting its conserved mechanisms for cellular protection during stress in all kingdoms. Interestingly, SnRK1 is activated by sugar depletion and under conditions of energy deficit, including darkness and hypoxia [57]. Once activated, SnRK1/Snf1/AMPK upregulates catabolism and downregulates anabolism to maintain energy homeostasis. This evidence suggests that ABA-induced SnRK1 activation signals low energy and carbon levels, which might condition to those signaled by trehalose [58]. Consistent with observation in our study, the trehalose contents (neg_9892) were accumulated in the rice CS tolerance line (Table S4), together with higher expression of genes related to SnRK1 and MAPK signaling and higher activities of antioxidant enzymes (Figure 4 and Figure 6C and Figures S2 and S5), suggesting that the SnRK1, MAPK4, and trehalose collaboratively work in CS response in rice.

## 4. Materials and Methods

### 4.1. Plant Materials and Growth Conditions

For the preliminary test, 128 rice varieties were used to screen for CS response. Finally, two distinct rice varieties, based on empty seed rates under CS against CK, were selected for this study, the resilient genotype Longdao18 (LD18) and the susceptible cultivar L9, focusing on CS tolerance during the vegetative stage. All rice varieties were obtained from the Institute of Crop Cultivation and Tillage at the Heilongjiang Academy of Agricultural Sciences. The plants were first grown in a growth chamber under a consistent temperature of 25 °C and a 12 h photoperiod until CS treatments were performed. In addition, a control temperature of 30 °C was implemented in a growth chamber condition to validate their performance under CS at the seedling and heading stages.

### 4.2. Cold Stress Treatments

Two stages (seedling stage and heading stage) were conducted for CS treatments. Forty seeds were sown into each dish for 10d and then exposed to CS conditions within a Conviron PGV36 walk-in cold growth chamber for 10d at 10 °C during the seeding stage. Regarding CS treatment during the heading stage, plants were raised in pots until reaching 30d of growth. Subsequently, the potted plants were exposed to CS conditions 10d at 15 °C. The growth chamber maintained optimal conditions at 75–85% relative humidity (RH), 800 μmol s^−1^m^−2^ light intensity positioned above 60 cm from the floor and 12 h photoperiod. After CS treatments, empty seed rates were used as the main parameter to screen the distinct CS response.

### 4.3. Photosynthetic Measurements

We employed a portable photosynthesis measurement system, the Licor 6400XT (LICOR Corp., Waltham, NA, USA), to conduct photosynthetic measurements. Following established protocols, this system evaluated photosynthetic rates (*A*) and stomatal conductance (g_s_) [59]. The system operated at a flow rate of 400 mmol s^−1^ for CO_2_ while maintaining light density at 1500 μmol m^−2^ s^−1^ and a temperature of 27 °C, with CO_2_ levels set at 400 ppm. The experiment comprised four biological replicates to ensure the strength and reliability of the obtained data.

### 4.4. Agronomic Traits

The CS treatment in this study was carried out during the booting stages of rice. Five representative plants were selected for the treatment to determine various agronomic traits. These included assessing the number of whole and empty grains per panicle and quantifying the number of deflated grains.

### 4.5. Antioxidant and Oxidoreductase Measurements

GR activity [60], soluble protein content [61], and SOD activity (measured by inhibiting the photoreduction of nitro blue tetrazolium (NBT) with modifications) [60] were assessed. The reaction mixture, totaling 3 mL, was composed of 25 mM phosphate buffer (pH 7.8), 13 mM methionine, 75 mM NBT, 0.1 mM EDTA, 4 µm riboflavin, 0.25 mL distilled water, and 0.05 mL enzyme extract. Initiated by riboflavin addition, the glass test tubes were exposed to fluorescent lamps (60 µmol m^−2^ s^−1^) for 20 min, stopped by turning off the light. One unit of activity is defined as the enzyme amount inhibiting 50% of NBT photoreduction at 560 nm. The determination of GSH levels was carried out with a modified version of an established protocol [62]. Fresh leaves and roots (0.25 g) were homogenized in 2.5 mL of ice-cold 5% (*w*/*v*) 5-sulfosalicylic acid, utilizing a chilled mortar and pestle. The homogenate was then centrifuged at 20,000× *g* for 20 min at 4 °C. CAT activity measurement relied on the reaction solution (3 mL) of 56 mm H_2_O_2_ and 0.2 mL enzyme extract, with absorbance changes at 240 nm recorded every 30 s defining activity as µmol H_2_O_2_ reduced/min/g protein [63]. POD activity, adapted from a previous protocol [63], was determined using a 3 mL solution comprising 0.1 mL enzyme extract and 2.6 mL of 0.3% guaiacol, initiated by adding 0.3 mL of 0.6% H_2_O_2_. The measurement of absorbance changes at 470 nm was performed every 30 s, defining activity as absorbance changes per minute and specific activity as enzyme units per gram of soluble protein. Glutamine synthetase [64], NAD-MDH, NADP-MDH [65], NAD-ME, and NADP-ME [66] activities were assessed.

### 4.6. Transmitted Electron Microscopic Analysis

As mentioned above, seven-day-old rice seedlings from L9 and LD18 were exposed to either CK or CS. Leaf tissues were collected and fixed for 24 h in sodium phosphate buffer at pH 7.2, containing 4% (*v*/*v*) glutaraldehyde and 3% (*w*/*v*) paraformaldehyde. Following fixation, the samples underwent three rinses, each lasting 20 min, in sodium phosphate buffer at pH 7.2. Subsequently, post-fixation was conducted for 1.5 h in sodium phosphate buffer (pH 7.2) supplemented with 1% (*v*/*v*) osmium tetroxide. Following this step, the samples underwent a dehydration process, initially using ethanol in the following sequence, 50%, 70%, and 90% for 10 min each, followed by two rounds of 100% ethanol for 15 min each. Afterward, they were dehydrated twice with 100% acetone for 15 min each. Following dehydration, the specimens were infiltrated and embedded in Epon-812 Resin. Ultrathin sections, measuring 50–70 nm, were prepared using a Leica EM UC 6 ultra-microtome. These sections were then mounted on copper grids and stained with 4% (*w*/*v*) uranyl acetate and lead citrate for 10 min. Subsequently, they were examined at 60–80 kV using a Zeiss EVO40 transmission electron microscope.

### 4.7. RNA Extraction and RNA Sequencing

Leaf samples from each group at the heading stage, either exposed to CS or CK, were obtained from three distinct replicates and individually placed in an RNA-stabilizing solution. To maximize the inclusion of plants in a single biological replicate, 1–2 leaves were collected from each seedling. These three biological replicates encompassed distinct sets of CS treatments. Around 80 mg of this powdered tissue was used for mRNA extraction using the XcelGen total RNA isolation kit (Xcelris Genomics, San Diego, CA, USA), followed by RNA sequencing analysis. The purity values and integrity of the isolated RNA were assessed by measuring with the Nanodrop 8000 Spectrophotometer (Thermo scientific, Waltham, MA, USA) and RNA 6000 Nano LabChip on the Agilent Bioanalyzer 2100 (Agilent Technologies, San Diego, CA, USA), respectively. Sequencing libraries were prepared using the Illumina TruSeq RNA Library Preparation Kit protocol (Illumina, San Diego, CA, USA). RNA-Seq was performed using the Illumina HiSeq2000 platform (Illumina, San Diego, CA, USA).

Genomic data for the reference cultivar, Nipponbare (*Oryza sativa* L. subsp. *japonica*), were retrieved from the publicly available repository at ftp://ftp.plantbiology.msu.edu/pub/data/EukaryoticProjects/osativa/annotationdbs/pseudomolecules/version_7.0/all.dir/; accessed on 14 January 2021. Transcriptome libraries from all the samples were aligned to the rice Nipponbare genome using TopHat (v1.4.3) and Bowtie (v0.13.8) with default parameters. For differentially expressed gene (DEG) analysis, Cufflinks (v1.3.3) was employed, generating FPKM values through reference-guided mapping [67]. Genes expressing notably low levels were omitted from the DEG analysis. DEGs were identified at a false discovery rate (FDR) of 0.05 and a *p*-value of ≤0.05. Heat maps illustrating differentially expressed genes were generated using the R package ’CummeRbund’ [68].

### 4.8. Gene Ontology and KEGG Analysis

Gene Ontology analysis of the DEGs was conducted using the agriGo toolkit and database. Statistical methods, including the Hypergeometric test, were employed to identify significant GO terms with a threshold of *p*-value <  0.05. BLAST comparisons were performed against the Kyoto Encyclopedia of Genes and Genomes (KEGG) GENES database to functionally annotate the DEGs using the KEGG Automatic Annotation Server (KAAS). The assignment of KEGG terms utilized the single-directional best hit (SBH) option, considering the representative gene data set for rice, specifically *Oryza sativa* Ref. Seq. Pathway mapping was achieved using the KEGG Orthology database, https://www.genome.jp/kegg/ko.html; accessed on 18 June 2021 [69].

### 4.9. Quantitative Transcript Measurements

To validate the expression of DEGs identified in response to CS in L9 and LD18, qPCR was used following transcriptome analysis. The RNA samples used corresponded to those employed in the RNA sequencing analysis. The RNA extraction and reverse transcription to cDNA were conducted following established procedures [70]. For qPCR analysis, SYBR Green PCR Master Mix (Yuanye Bio, Shanghai, China) was utilized with a real-time PCR system (ABI StepOnePlus, Applied Biosystems, USA). Primers used for qPCR are listed in Table S1. The running program for qPCR included 95 °C for 30 s, followed by PCR with 45 cycles at 95 °C for 15 s, 61 °C for 20 s and 72 °C for 30 s. Each assay was conducted with three biological samples. The housekeeping gene is actin1. Relative gene expression against the housekeeping gene was calculated using the 2^−ΔΔCT^ method (ΔCT  =  CT, gene of interest^−CT^) [71].

### 4.10. Metabolism Determinations

For metabolite determinations, samples were promptly harvested upon completion of the CS treatments in both L9 and LD18 rice lines. Samples were taken from the marked leaf sections used for gas exchange measurements. Non-targeted metabolic profiling of the leaves from LD9 and LD18 under control and CS was conducted using the LC-MS/MS (Triple Quad 6500, SCIEX, Goleta, CA, USA) [70]. Approximately ~3.0 mg leaf samples from the LD9 and LD18 subjected to CS were collected in re-cooled 2 mL Eppendorf tubes and promptly stored in liquid nitrogen. The samples underwent initial extraction using a ball mill at 50 Hz for 10 min. The extracted powder was then dissolved in a 1.5 mL methanol/chloroform mixture and incubated at −20 °C for 12 h. Subsequently, the mixture was centrifuged at 2500× *g* and 4 °C for 15 min and filtered using 0.43 μm organic phase medium (GE Healthcare, 6789–0404).

We performed metabolic analysis utilizing Metabolon software (Durham, NC, USA) and identified sample components according to retention time and mass spectra with reference metabolites. For precise identification of metabolic compounds in each sample, it is strongly advised to consult the mass spectra with entries in the NIST02 and metabolome database (http://gmd.mpimp-golm.mpg.de, accessed on 20 January 2025).

## Data Availability

All data are available in the manuscript or the Supplementary Materials. All related sequencing data are deposited in the NCBI Sequence Read Archive (SRA) database with the link https://www.ncbi.nlm.nih.gov/sra?term=PRJNA793928 (accessed on 25 January 2025). The bioProject accession is PRJNA793928.

## Supplementary Materials

The following supporting information can be downloaded at: https://www.mdpi.com/article/10.3390/plants14040498/s1, Figure S1: Spikelet traits of LD18 and L9 rice lines exposed to 20-d CS at the graining stage; Figure S2: Activity of antioxidant changes in the leaves of LD18 and L9 exposed to CS conditions during heading stage; Figure S3: Activities of enzymes involved in carbon assimilation and energy metabolism in LD18 and L9 under CS during heading stage; Figure S4: GO analysis on the DEGs in L9 and LD18 under CS relative to CK; Figure S5: qPCR validation of upregulated DEGs in LD18 due to CS effects; Figure S6: Non-targeted metabolic analysis of DAMs in LD18 and L9 under CS relative to CK; Figure S7: Extremely DAMs in either L9 or LD18 under CS relative to CK; Table S1: Primers used in this study; Table S2: Statistical analysis on the reads based on transcriptome analysis; Table S3: List of top 1% DEGs in LD18 with up-regulated in CS, while no significant or upregulated in L9 in CS; Table S4: Top DAMs in LD18 relative to L9 in CS relative to CK; Data S1: Numbers of empty and full spikelet in 128 rice accessions exposed to cold stress used in this study; Data S2: FPKM values of differentially expressed genes in L9 and LD18 exposed to either CK or CS based on transcriptome analysis.
