# Supplementary material for: Identifications of Genes Involved in ABA and MAPK Signaling Pathways Positively Regulating Cold Tolerance in Rice
Source: Plants (Basel). 2025 Feb 7;14(4):498. doi: 10.3390/plants14040498 (PMC11859393; doi:10.3390/plants14040498)
Supplement: Supplementary file 1 [file plants-14-00498-s001.zip › Supplemental Figures.pdf]

Supplemental Figures

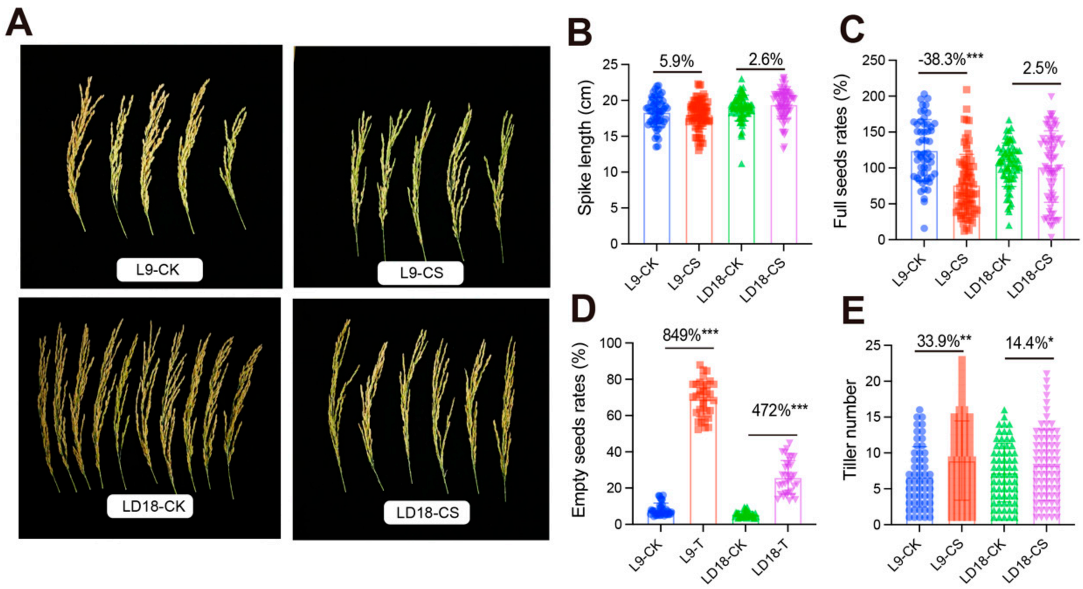

Figure S1. Spikelet traits of LD18 and L9 rice lines exposed to 20-d CS at the graining stage. **A**, Images of spikelet derived from each plant for each rice line. **B-E**, Spike length, full seeds rates, empty seed rates, and tillering. Photosynthetic rates and stomatal conductance under statured light conditions in the leaves of LD18 and L9. Vertical bars represent means ± S.E. (*n*=30). Percentage differences in CS compared to CK for each rice line. Symbols "*", "**" and "***" indicate significant differences at *P*<0.05, *P*<0.01 and *P*<0.001, respectively, based on Student *t*-test analysis.

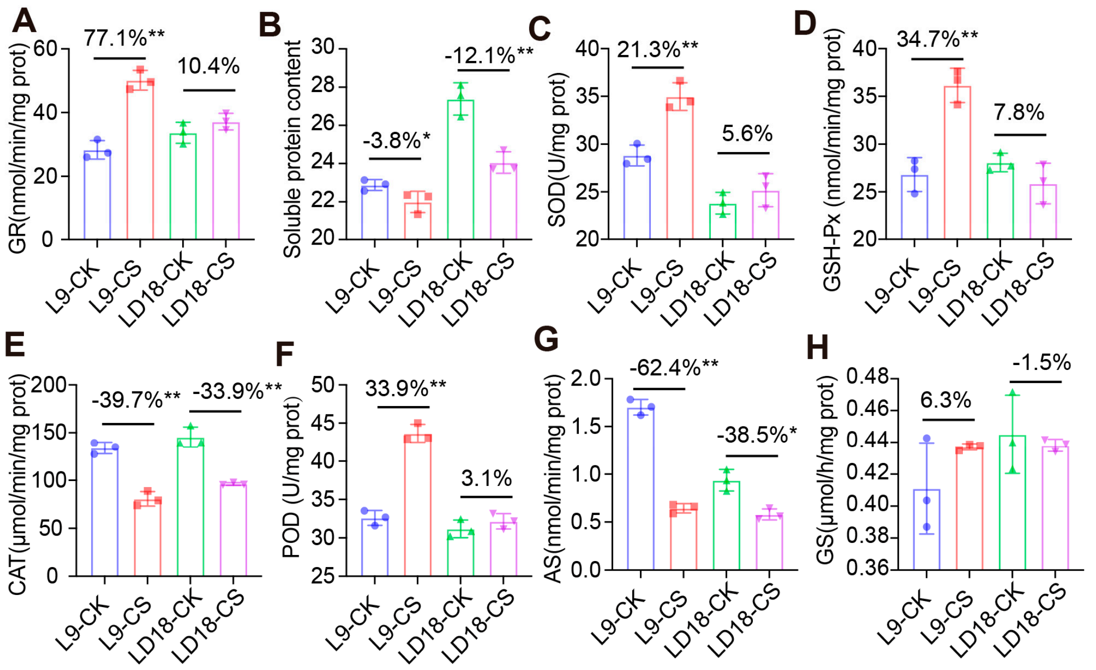

Figure S2. Activity of antioxidant changes in the leaves of LD18 and L9 exposed to CS conditions during heading stage. **A,** GR (Glutathione reductase). **B,** Soluble protein content. **C,** SOD (superoxide dismutase). **D,** GSH-Px (reduced glutathione). **E,** CAT (catalase). **F,** POD (peroxidase). **G,** AS (asparagine synthase). **H,** GS (glutamine synthetase). Vertical bars represent as means ± S.E. (n=3). Percentage differences in CS compared to the CK for each rice line are presented. Symbols "*", "**" and "***" indicate significant differences at P<0.05, P<0.01 and P<0.001, respectively, based on Student t-test analysis.

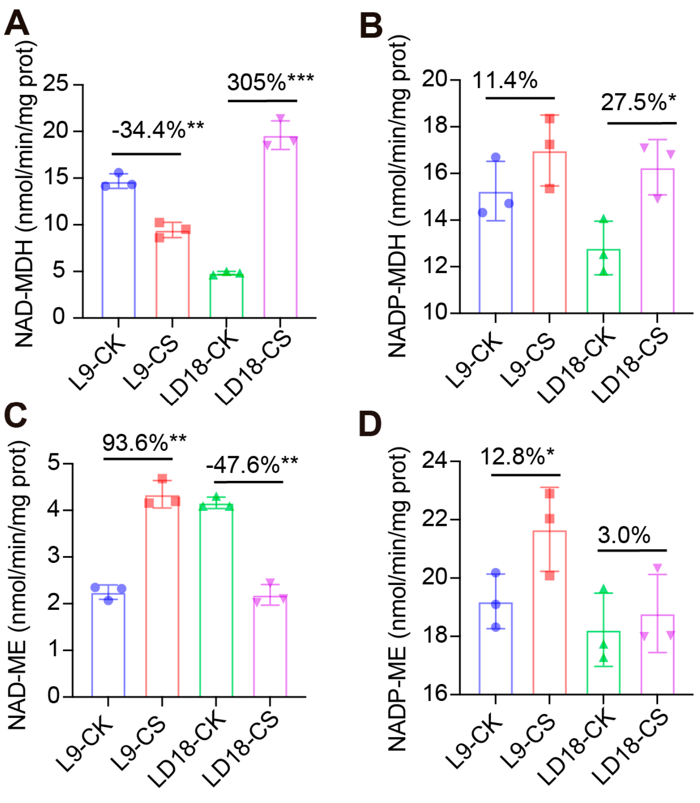

Figure S3. Activities of enzymes involved in carbon assimilation and energy metabolism in LD18 and L9 under CS during heading stage. **A**, NAD-MDH; **B,** NADP-MDH. **C,** NAD-ME; **D,** NADP-ME. Vertical bars represent means ± S.E. (*n*=3). Percentage differences in CS against the CK for each rice line. Symbols "*", "**" and "***" indicate significant differences at *P*<0.05, *P*<0.01 and *P*<0.001, respectively, based on Student *t*-test analysis.

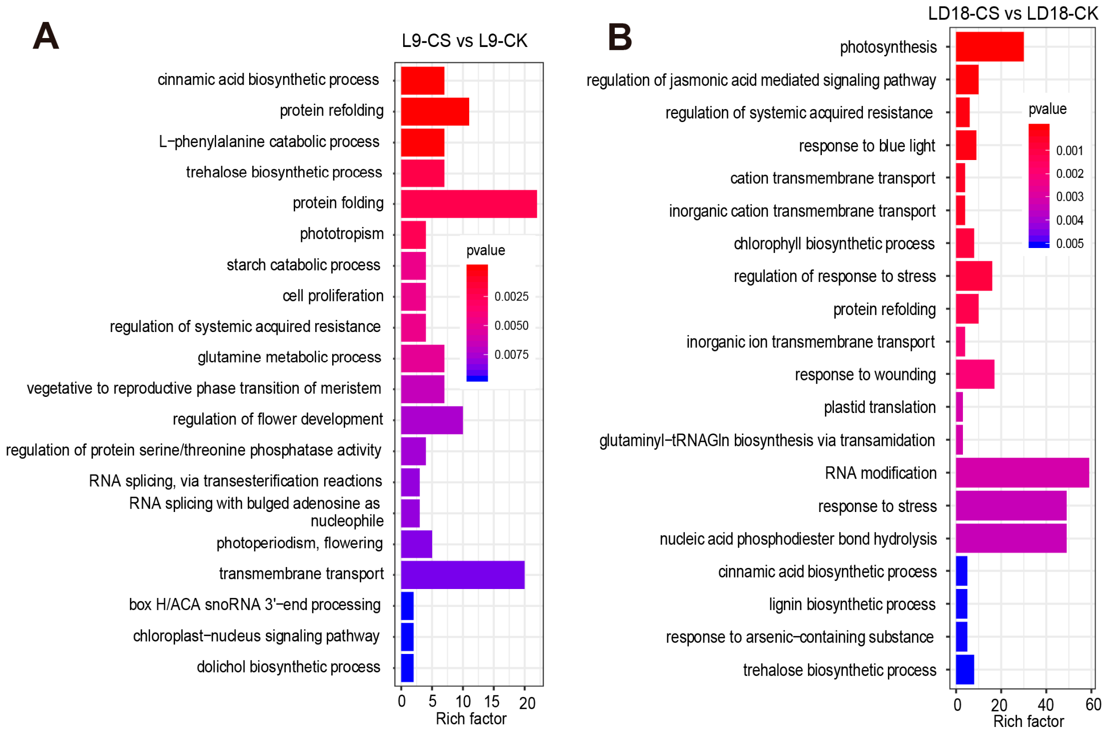

Figure S4. GO analysis on the DEGs in L9 and LD18 under CS relative to CK. **A**, GO analysis on the DEGs in L9 under CS compared to CK. **B**, GO analysis on the DEGs in LD18 under CS compared to CK.

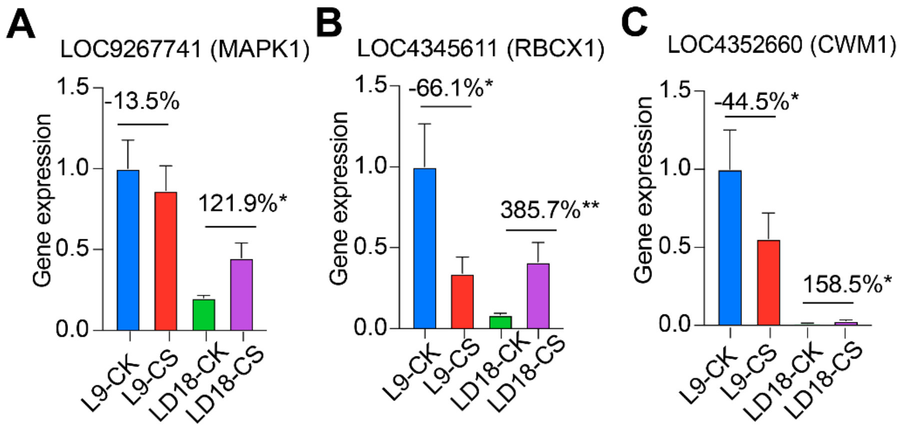

Figure S5. qPCR validation of upregulated DEGs in LD18 due to CS effects. **A-C,** Relative expression of genes, including *LOC9267741*(*MAPK1*), *LOC4345611* (*RBCX1*) and *LOC4352660* (*CWM1*). Vertical bars represent means ± S.E. (n=3). Percentage differences CS compared to CK for each rice line. Symbols “*”, “**”

and "***" indicate significant difference at *P*<0.05, *P*<0.01 and *P*<0.001, respectively, based on Student *t*-test analysis.

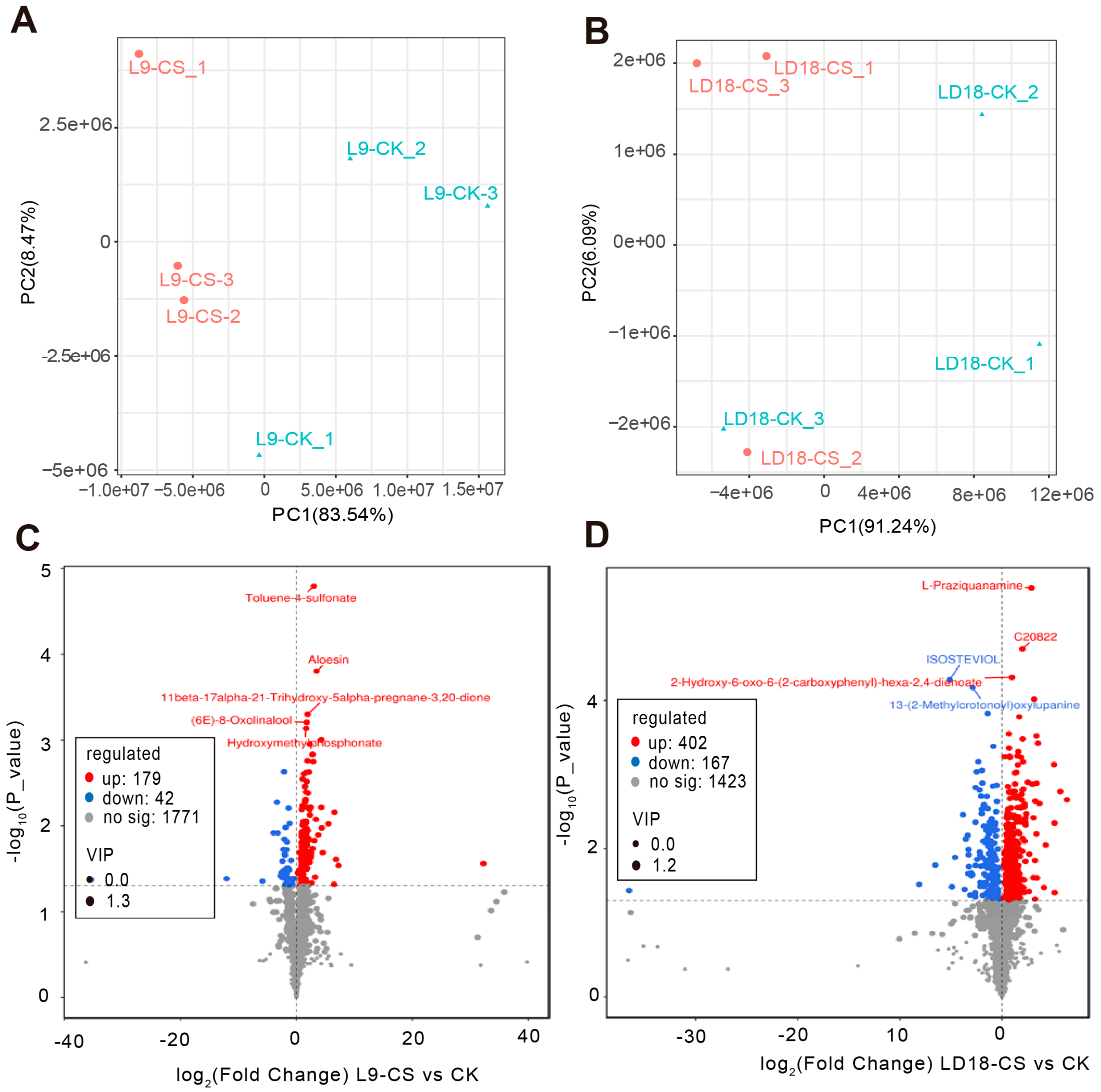

Figure S6. Non-targeted metabolic analysis of DAMs in LD18 and L9 under CS relative to CK. **A-B,** PCA of DAMs in L9 (**A**) and LD18 (**B**) under CS compared to CK. **C-D,** Volcano plot representing DAMs in L9 (**C**) and LD18 (**D**) under CS relative to CK. The names of extremely DAMs were labeled in figures **C-D**.

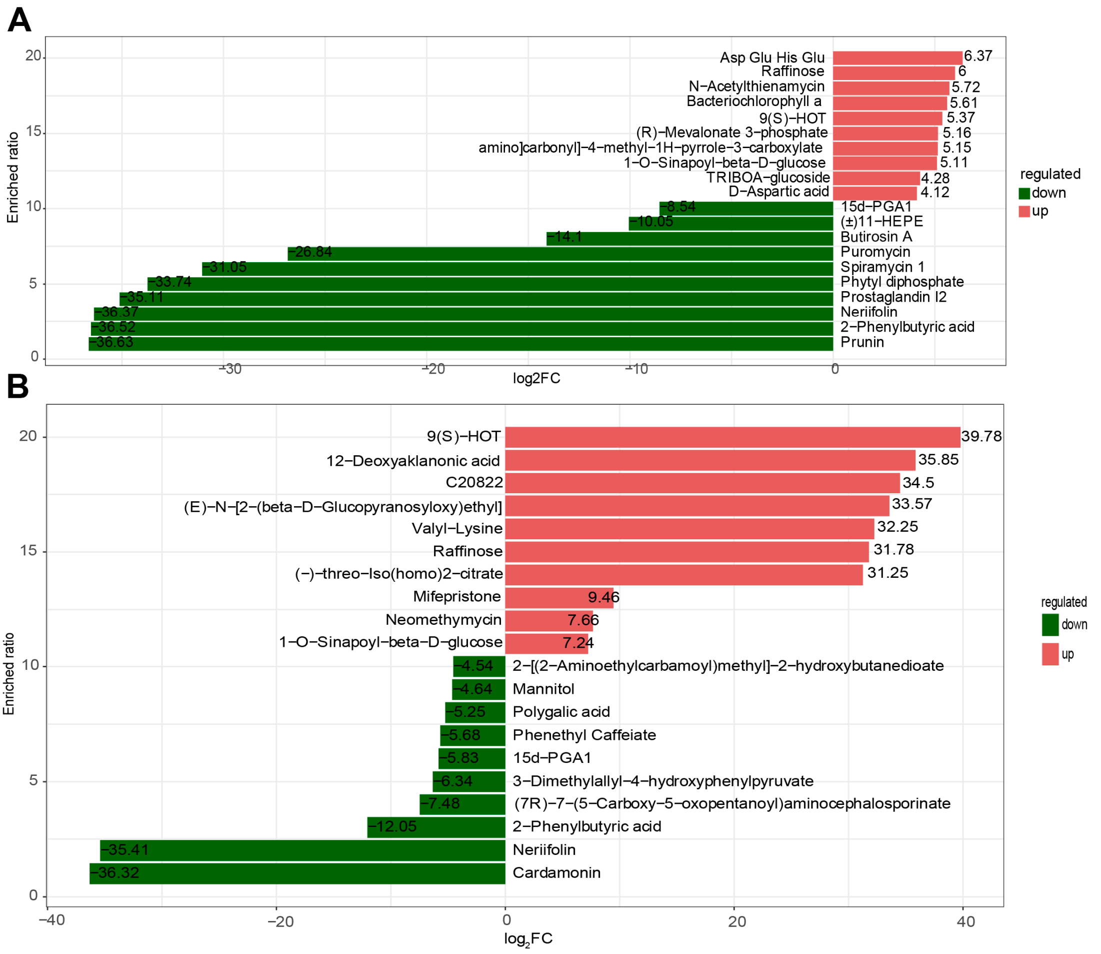

Figure S7. Extremely DAMs in either L9 or LD18 under CS relative to CK. **A**, Extremely DAMs in L9. **B**, extremely DAMs in LD18. The data are referred to Figure S4C-D.
