# Supplementary material for: Identifications of Genes Involved in ABA and MAPK Signaling Pathways Positively Regulating Cold Tolerance in Rice
Source: Plants (Basel). 2025 Feb 7;14(4):498. doi: 10.3390/plants14040498 (PMC11859393; doi:10.3390/plants14040498)
Supplement: Supplementary file 1 [file plants-14-00498-s001.zip › Supplementary Tables.pdf]

**Supplementary Tables**

**Table S1.** Primers used in this study.

| Gene ID | sequence (5'to3') |
|---|---|
| 18S-F | CTACGTCCCTGCCCTTTGTACA |
| 18S-F | ACACTTCACCGGACCATTCAA |
| LOC4327351-F | ATTAGCCATCAACGACCA |
| LOC4327351-R | ACTCTCCAATCTGCTCTTC |
| LOC4331490-F | TGGCACAACTTACCTCTG |
| LOC4331490-R | CGCACATCTTCTACATCCT |
| LOC4331855-F | TGTGTTGCTGTGTTCTGG |
| LOC4331855-R | AGGAATGATGGGCTTTGC |
| LOC4332786-F | CCAAGAAGAGGCATGTGTT |
| LOC4332786-R | AAATCTTTCCAGCCAGCAA |
| LOC4333690-F | AACTGGAGGAACTGATTGG |
| LOC4333690-R | CACTGGACACGATAACGA |
| LOC4335640-F | GTCCCTTTACGACAATGGT |
| LOC4335640-R | CACCCGTGCTATCCATTAG |
| LOC4337850-F | CTACAGCCAAACCAAGGA |
| LOC4337850-R | ATCGGTGCTTCTCATACG |
| LOC4345611-F | GAGGTCCGATCTGCTTATG |
| LOC4345611-R | GCTGGTCTCCAAGACATAG |
| LOC4349417-F | TGAGCAGGCATCATGTCTAG |

| | |
|---|---|
| LOC4349417-R | ATGGTGGGCAGTTTACACAT |
| LOC4352460-F | ATGGCTTGTAGAGTAATGCT |
| LOC4352460-R | CTCTGCTGATCTGAACTCAT |
| LOC4352660-F | CTTGTTTCAGAGCAGGTA |
| LOC4352660-R | GCAGTATGGTTCATCAGA |

**Table S2.** Statistical analysis on the reads based on transcriptome analysis.

| Sample name | Clean reads | Clean bases | GC Content | %≥Q30 |
|---|---|---|---|---|
| L9_CK_1 | 21793809 | 6414488540 | 0.5239 | 0.9446 |
| L9_CK_2 | 22146015 | 6515861142 | 0.5281 | 0.943 |
| L9_CK_3 | 21773902 | 6412091398 | 0.5259 | 0.9442 |
| L9_CS_1 | 19561507 | 5773695086 | 0.5316 | 0.945 |
| L9_CS_2 | 22755703 | 6719458346 | 0.5306 | 0.9453 |
| L9_CS_3 | 20774756 | 6074553996 | 0.5263 | 0.9493 |
| LD18_CK_1 | 19839480 | 5819327142 | 0.5276 | 0.9489 |
| LD18_CK_2 | 23451821 | 6896476236 | 0.5166 | 0.9419 |
| LD18_CK_3 | 21757955 | 6442497112 | 0.5262 | 0.9288 |
| LD18_CS_1 | 19282306 | 5712842136 | 0.5303 | 0.9387 |
| LD18_CS_2 | 21248139 | 6283679780 | 0.5284 | 0.9392 |
| LD18_CS_3 | 22057167 | 6490292348 | 0.5252 | 0.9329 |

**Table S3.** List of top 1% DEGs in LD18 with up-regulated in CS, while no significant or upregulated in L9 in CS.

| Gene ID | Log2Fold change (LD18_CS vs CK) | Log2Fold change (L9_CS vs CK) | Pathway | Abbreviation |
|---|---|---|---|---|
| gene-*LOC4333690* | 2.10 | no sig. | ABA | *GRP3* |
| gene-*LOC4345611* | 3.29 | no sig. | Photosynthetic capacity | *RBCX1* |
| gene-*LOC4331855* | 11.93 | 2.85 | CS response | *CRP27* |
| gene-*LOC9269592* | 1.64 | 2.06 | N.A. | *RSBC3* |
| gene-*LOC4330101* | 1.31 | no sig. | MAPK | *CRP1* |
| gene-*LOC4331490* | 1.24 | no sig. | MAPK | *CDPK 11* |
| gene-*LOC4349417* | 4.60 | 6.57 | Carbon metabolism | *AMY3* |
| gene-*LOC4332786* | 1.64 | no sig. | MAPK | *STK1* |
| gene-*LOC4352660* | 5.18 | 3.16 | MAPK | *CWM1* |
| gene-LOC9267741 | 1.71 | no sig. | MPK1 | *MAPK1* |
| gene-LOC4342267 | 3.11 | no sig. | MAPK | *MAPK4* |
| gene-LOC4342017 | 1.77 | no sig. | MAPK | *MAPK2* |
| gene-*LOC4335640* | 2.91 | 1.32 | ABA | *SnRK2* |
| gene-*LOC4339173* | 2.36 | no sig. | ABA | *SnRK1* |
| gene-*LOC4336878* | 0.71 | no sig. | CS response | *COLD1* |

| | | | | |
|---|---|---|---|---|
| gene-*LOC4330018* | 0.97 | no sig. | MAPK | *LTG1* |
| gene-*LOC4332475* | 0.34 | no sig. | MAPK | *OsMPK3* |

**Table S4.** Top DAMs in LD18 relative to L9 in CS relative to CK.

| #ID | Name | Regulated | L9_CK | L9-CS | LD18-CK | LD18-CS | HMDB_taxonomy |
|---|---|---|---|---|---|---|---|
| neg_751 | Fumarate | up | 39.1688 | 144.73 | 18.2436 | 174.577 | Organooxygen compounds |
| neg_13906 | Methyl jasmonate | up | 1429.04 | 7184.87 | 527.776 | 5562.45 | Fatty Acyls |
| neg_9892 | ALPHA,BETA-TREHALOSE | up | 300.986 | 1452.87 | 156.642 | 1401.86 | -- |
| neg_11285 | 5,6,7,8-Tetrahydromonapterin | up | 16.7275 | 49.1205 | 50.0153 | 247.266 | -- |
| neg_11948 | 4-Hydroxy-3-methoxyphenyl-beta-hydroxypropanoyl-CoA | up | 91.2738 | 229.441 | 96.7683 | 389.258 | -- |
| neg_7335 | 3-Hydroxypicolinic acid | down | 22.678 | 2.46515 | 35.8062 | 10.8276 | -- |
| neg_7815 | 4-Nitrophenol | down | 615.554 | 99.1333 | 900.92 | 239.777 | Benzene and substituted derivatives |
| neg_4695 | Indole-3-carboxylic acid | down | 50.1827 | 13.6965 | 63.0731 | 26.388 | Indoles and derivatives |
| neg_8162 | Aclacinomycin A | down | 619.369 | 95.0861 | 570.001 | 124.314 | -- |
| neg_8887 | cis-4-Hydroxy-D-proline | down | 140.613 | 31.5766 | 183.115 | 56.7915 | Carboxylic acids and derivatives |
